# Breaking free from tunnel vision for climate change and health

**DOI:** 10.1371/journal.pgph.0001684

**Published:** 2023-03-09

**Authors:** Thilagawathi Abi Deivanayagam, Rhiannon Elizabeth Osborne

**Affiliations:** 1 Institute for Global Health, University College London, London, United Kingdom; 2 Lancaster Medical School, Faculty of Health and Medicine, Lancaster University, Lancaster, United Kingdom; 3 School of Clinical Medicine, Addenbrooke’s Hospital, University of Cambridge, Cambridge, United Kingdom; McGill University, CANADA

## Abstract

Climate change is widely recognised as the greatest threat to public health this century, but ‘climate change and health’ often refers to a narrow and limited focus on *emissions*, and the *impacts* of the climate crisis, rather than a holistic assessment of economic structures and systems of oppression. This tunnel vision misses key aspects of the climate change and health intersection, such as the enforcers of planetary destruction such as the military, police, and trade, and can also lead down dangerous alleyways such as ‘net’ zero, overpopulation arguments and green extractivism. Tunnel vision also limits health to the absence of the disease at the individual level, rather than sickness or health within systems themselves. Conceptualising health as political, ecological, and collective is essential for tackling the root causes of health injustice. Alternative economic paradigms can offer possibilities for fairer ecological futures that prioritise health and wellbeing. Examples such as degrowth, doughnut economics and ecosocialism, and their relationship with health, are described. The importance of reparations in various forms, to repair previous and ongoing harm, are discussed. Breaking free from tunnel vision is not simply an intellectual endeavour, but a practice. Moving towards new paradigms requires movement building and cultivating radical imagination. The review highlights lessons which can be learnt from abolitionist movements and progressive political struggles across the world. This review provides ideas and examples of how to break free from tunnel vision for climate change and health by highlighting and analysing the work of multiple organisations who are working towards social and economic transformation. Key considerations for the health community are provided, including working in solidarity with others, prioritising community-led solutions, and using our voice, skills, and capacity to address the structural diagnosis—colonial capitalism.

## Introduction

**Fig 1:**
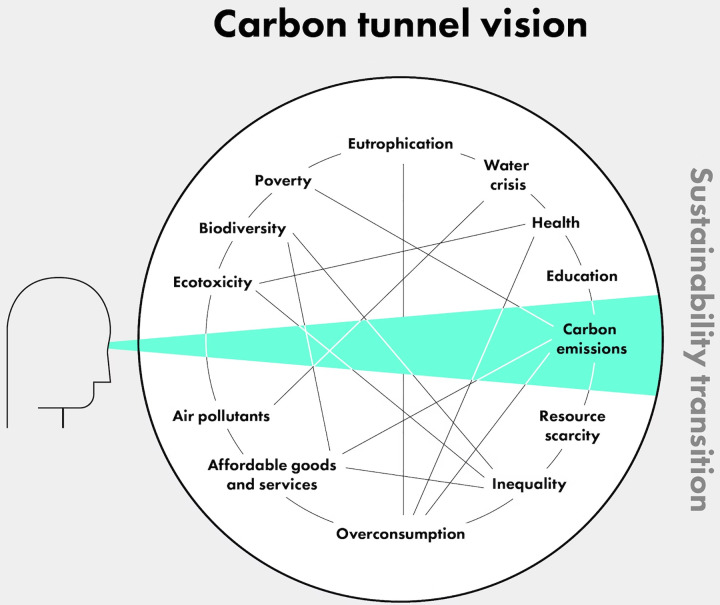
Carbon tunnel vision [2].

“Climate change is the greatest threat to human health this century” [1]. This is the foundation of the mainstream climate change and health movement, which has been trying to accelerate mitigation and adaptation efforts on climate change using the lens of health. It is a powerful frame but is ultimately akin to starting our story from the penultimate chapter. Focusing on the health *impacts* of climate change has generated a degree of tunnel vision, where the story of how we got here is neglected in favour of a limited analysis focussed on emissions, a downstream indicator of climate change [See Fig 1] [2].

Long before health professionals in the global North acknowledged the harms of air pollution or rising temperatures on health, communities at the frontline of the fossil fuel industry raised the alarm. Life expectancy in the Niger Delta, home to projects by the oil giant Shell, is reported to be 10 years lower than the rest of Nigeria [3]. Communities in the region have spent decades documenting the health harms and resisting the expansion of oil extraction and have collectively developed a manifesto for repairing the health and ecological violence inflicted by the industry [4]. Shell itself is an example of a colonial project. In 1956, prior to Nigeria’s independence, Shell convinced the Nigerian government to create a joint venture, which began oil exploration in the Niger Delta, asserting corporate colonial control over the land [5]. Fossil fuel extraction causes death and sickness globally, from undrinkable water due to Chevron’s pollution in the Amazon, to the racialised impact of fracking in the US [6]. Communities near sites of extraction face violence as corporations, aided by governments, seize the ‘natural resources’. Land defenders are murdered and criminalised [7], and thousands are killed in wars to facilitate oil access for multinational companies [8].

Climate change is a product of, and can interact with, various oppressive systems, such as patriarchy, white-supremacy, class oppression, and ableism [9]. These systems of oppression designate the lives of some communities as disposable, enabling and justifying the erosion of their health for the extractive economy [10]. For example, white supremacy enables resource theft, deems large parts of the globe climate sacrifice zones, forces certain communities to live in pollution hotspots, and deports refugees fleeing the climate disasters it creates. Class oppression enables excessive corporate power whilst eroding trade unions, leading to further destruction and resource capture [11].

“In a global economy predicated on the extraction of resources and the relentless pursuit of profit, systems of oppression–white supremacy, patriarchy, ableism, neoliberal capitalism–simultaneously drive health inequalities and climate change.”—Global Health Watch 6 [12]

Systemic harms imposed on people and the planet are not unique to the fossil fuel industry, but a pattern across extractive industries fuelling the climate and ecological crises, such as the fast fashion and agribusiness industries [13]. Each of these industries make profit through exploiting the working classes, in particular racialised people, people oppressed due to their gender, and disabled people, while also destroying the fragile web of life maintained by all beings on this planet. As Voskoboynik emphasises, “ecocide came hand in hand with ethnocide” [14]. Meanwhile governments facilitate corporate globalisation and the billionaire ruling class controlling markets and resources, resulting in deepening of health and social inequalities. So long as there is deregulation of markets and growth of neoliberalism, corporations and governments will continue to appropriate, privatise, and commodify the planet’s resources and its people [13].

To address the health inequities rooted in these structures, the climate crisis must be understood as the consequence of capitalist plunder [15]. Colonialism, a world order built on power hierarchies, has always been a project of extracting vast amounts of ‘natural resources’ and labour from colonised countries in order to fuel ‘economic growth’ in the coloniser countries. Capitalism requires the exploitation of people and nature to generate profit and wealth accumulation only for elites [16]. This concentrates resources in the hands of rich elites and corporations, whilst dumping the waste from extractive industries onto poor and marginalised communities and denying the majority of people the essentials of a decent life.

*“Do we choose industry*, *or do we choose life*?*”—Kanahus Manuel*, Secwepemc and Ktunaxa

Colonial plunder meant that Indigenous Peoples were killed for their land while the coloniser pursued mining and other extractive enterprises. Instead of a profitable resource to be exploited, Indigenous practice sees land and ecological systems as inseparable from health [17]. Harming land and ecology, the fabric of communities, means harming health. Planetary health in its broadest and decolonial sense should be grounded in an ecocentric approach that develops understandings and seeks solutions from Indigenous values, worldviews, and knowledge systems [18]. Yet, these framings of health are seen as utopian, and calls to transform the economic system at the root of the health impacts of climate change is deemed too radical.

Facilitated by violence, rigged trade law, and deliberate impoverishment, rich countries have drained $152tn from the global South since 1960; this is neocolonialism [19]. The global North is responsible for 92% of excess emissions and the majority of ecological breakdown [20]; 100 companies account for 70% of all emissions [21]; and the richest 1% of people globally are responsible for more emissions than the bottom 50% [22].

Birthed from world-systems theory [23], global North refers to the “core” economically powerful countries, whereas global South refers to “peripheral” countries [23] that tend to be previously colonised societies that remain socially and culturally minoritised [24]. This language has its deficiencies. Not all people in the global North are more economically powerful, just as not everyone in the global South experiences struggle. Oppression by the elite bourgeoisie is shared by minoritised people all over the world, across multiple realities [25]. The climate and ecological crises can therefore not be separated from the elites’ wealth accumulation at the expense of the health, rights, and dignity of the global majority, which includes many people within rich countries themselves. Therefore, our solidarity must transcend the boundaries of geography, time, and nature of struggle.

This review is produced from collective practices, knowledge generation, and searching of sources and discourse across the health justice and climate justice movement spaces and beyond traditional academic literature. The ideas generated build on the rich body of work by those who came before us and work alongside us. As authors, we position ourselves as young health workers living in the global North, activists working in relation and solidarity with communities across the global South and people from Tamil, Welsh, and Armenian heritage.

## The enforcers of planetary destruction

Carbon tunnel vision focuses on emissions, not the systems and structures which are actively protecting and promoting climate destroying industries. Destructive and violent industries require enforcement; governments across the world consistently use state violence to do this.

The police and military are regularly deployed across the world to maintain the interests of fossil fuel companies and other extractive industries. Police violence against Standing Rock protestors in North Dakota [26], mostly Indigenous Peoples, included water cannons, stinger rounds, teargas grenades, tasers, and other life-threatening violence. Despite the violence facing them, resistance of Indigenous communities to extractive industries has averted emissions equivalent to at least 25% of the US and Canada’s annual emissions [27]. The clampdown on the right to protest in the UK during ecological collapse is no coincidence—investigations by OpenDemocracy revealed that the new policing bill was dreamed up by a right-wing think tank funded by ExxonMobil [28].

Beyond protecting fossil fuel interests, the police use violence to disproportionately target poor people, black people and marginalised people so much so that policing can be conceptualised as a threat to public health [29]. In addition, as highlighted by a recent letter in solidarity with Chris Kaba’s family, the racialised communities traumatised by police violence are the same ones affected by the worst air pollution and the climate crisis [30].

The oil motivations of the Iraq war have been extensively documented, including by Oil Change International—“Throughout the occupation, from 2003 to 2011, privatizing the oil–against the wishes of Iraqis–was a consistent U.S. priority, and was closely tied to military operations” [31]. Research by Greenpeace found that almost two-thirds of all EU military missions monitor and secure the production and transport of oil and gas to Europe. From 2018–2021 Italy, Spain and Germany invested over €4 billion to protect fossil fuels [32]. Militaries and oil companies collaborate extensively—evidence from witness statements and own company documents shows that Shell corporation encouraged the Nigerian military to suppress resistance to their operations [33]. In addition, militaries themselves are huge contributors to the climate and ecological crises. The US military accounts for 5% of annual global emissions [34] and military bases have been shown to be polluting water supplies with toxic ‘forever chemicals’ [35].

Another area of enforcement and protection of the interests of extractive industries is via global trade rules which ensure the protection of foreign investments, benefitting transnational corporations and enabling environmental destruction. For example, Investor State Dispute Settlements (ISDS) create bespoke legal arrangements for corporations [36]. The Energy Charter treaty (ECT), active since 1988, allows energy investors to file claims for ISDS, and is the world’s most litigated investor protection agreement [37]. In 2012, Ecuador lost an ISDS case against Occidental Petroleum, who had broken Ecuadorian law. Despite this, Ecuador was ordered to pay Occidental US$1.8 billion for terminating the contract—the equivalent of the country’s entire health budget [38]. In 2019, Infinito Gold sued Costa Rica over the introduction of a ban on open-cast mining for metals. The ECT is currently being used to sue governments for attempts to phase out fossil fuels, including The Netherlands and Germany.

These blind spots of tunnel vision to police violence, militarisation and global trade prevent us from tackling the enablers and enforcers of planetary destruction.

## The diagnosis is at the structural level

Without locating the diagnosis at the structural level, tunnel vision takes us down dangerous alleyways, including the overpopulation, ‘net-zero’ and green technology myths, leading to consequences that disproportionately affect minoritised people. These three case examples have been drawn from the authors’ experiences of working at this intersection, based in the global North.

### 1) Overpopulation

The overpopulation myth suggests that there are too many people in the world and insufficient resources. Often this argument suggests that people of reproductive age in ‘overpopulated’ countries (which mostly includes people of colour) should reduce their rate of births. Fabricated fears of overpopulation by certain groups are not new and have consistently been used to justify violent practices against minoritised groups, especially under settler colonialism and fascism [39]. Today, popular white environmentalists such as David Attenborough, and Jane Goodall, and Prince William, push for population control to tackle climate change and ‘resource scarcity’. On October 15th, the High Representative of the EU for Foreign Affairs and Security Policy, Josep Borrell Fontelles, said in a speech—“Europe is a garden…the rest of the world is a jungle…the jungle could invade the garden…the jungle has a strong growth capacity [40].”

Concern for overpopulation is often included alongside concern for access to sexual and reproductive health rights (SRHR). Access to family planning, abortion and other sexual and reproductive health services must be advocated for as a fundamental right, not as an initiative only worthwhile for a secondary purpose, such as a ‘solution’ to the climate crisis. Initiatives aimed at targeting ‘overpopulation’ have historically violated SRHR, including the use of forced sterilisation, usually targeted towards ‘undesirable’ social groups such as women of colour, people in poverty and disabled communities [41].

Propagating the myth of overpopulation has real life consequences. The White nationalist responsible for murdering 51 people in Christchurch, New Zealand, claimed in his manifesto, “Kill the invaders, kill the overpopulation, and by doing so save the environment [42]”. A gunman in Texas blamed overpopulation and another in Buffalo expressed a desire to kill as many Black people as possible, to act on environmental degradation. The overpopulation narrative on climate change is a weapon to disguise wealth hoarding by elites and scapegoat minorities. There is no evidence that countries with higher total human population growth rates have a greater environmental impact [43].

### 2) ‘Net-zero’

Though the concept may seem ambitious,‘net-zero’ enables climate inaction [44]. Carbon offsetting, tree planting and carbon capture—‘solutions’ that feature heavily in ‘net-zero’—are neither proven to be effective nor free from other forms of injustice. ‘Net-zero’ conveniently avoids targeting the true drivers of emissions, and allows companies like Shell and BP to declare they are ‘net-zero’ based on promised negative emissions in the distant future, whilst expanding fossil fuels [45]. Net-zero is a tool used by corporate powers and states to continue business as usual. The drivers of climate change are not challenged, and wealth continues to be accumulated by elites at the expense of the health of the majority.

It is also clear that a ‘net-zero’ health system that demands user fees in order to obtain healthcare from migrants and refugees whilst collaborating with border enforcement agents to identify people for deportation is not compatible with climate justice. Beyond the ‘net-zero’ field of vision, addressing health inequities created by the global arms trade, xenophobia, islamophobia, and violent border policies is part of the climate change and health intersection.

### 3) Green extractivism

Simply removing fossil fuels from an energy system rooted in extraction does not guarantee health equity. Whilst frontline global South communities have resisted fossil fuel extraction from global North corporations for decades, similar patterns are being replicated for green energy, from lithium for electric vehicles to solar and wind farms. For example, Indigenous Peoples’ right to consultation has been violated to pursue renewable projects conducted by EU companies, including the pursuit of the San Dionisio wind farms in Mexico [46].

Tunnel vision allows continuation of the violent human rights abuses when developing greener technology and energy. Beyond emissions, other social and environmental boundaries are crossed during this wave of green extractivism. A lens of health justice enables us to challenge corporate and colonial resource theft dressed in green.

“*Green is supposed to be the colour of life and the biosphere but*, *increasingly*, *green symbolises the market and money*, *and a green economy could entail the* ultimate commodification *of the planet” [13]—Vandana Shiva*

The vast health benefits of a renewable energy system can be fully realised through community owned, collectively governed systems, which centre global equitable access to resources and energy. As highlighted by the People’s Health Movement Ecosystem and Health Working Group, the entire extractive economy must be overturned to achieve health and environmental justice, and ‘Green New Deals’ for the global North which fail to address this are likely to create ‘green’ extractive colonialism [47]. Alternatives, such as the recently proposed *Manifesto for an Ecosocial Energy Transition from the Peoples of the South*, centre energy democracy and global justice [48].

## Tunnel vision for health

Trapped by tunnel vision, the chance to imagine radical solutions for sustainability is stolen from the collective imagination by those benefiting from the current economic system. Without understanding the climate crisis as a symptom of sick systems, it is easy to see emissions reductions as a ‘sacrifice’, rather than an opportunity to radically reshape the world.

**Fig 2:**
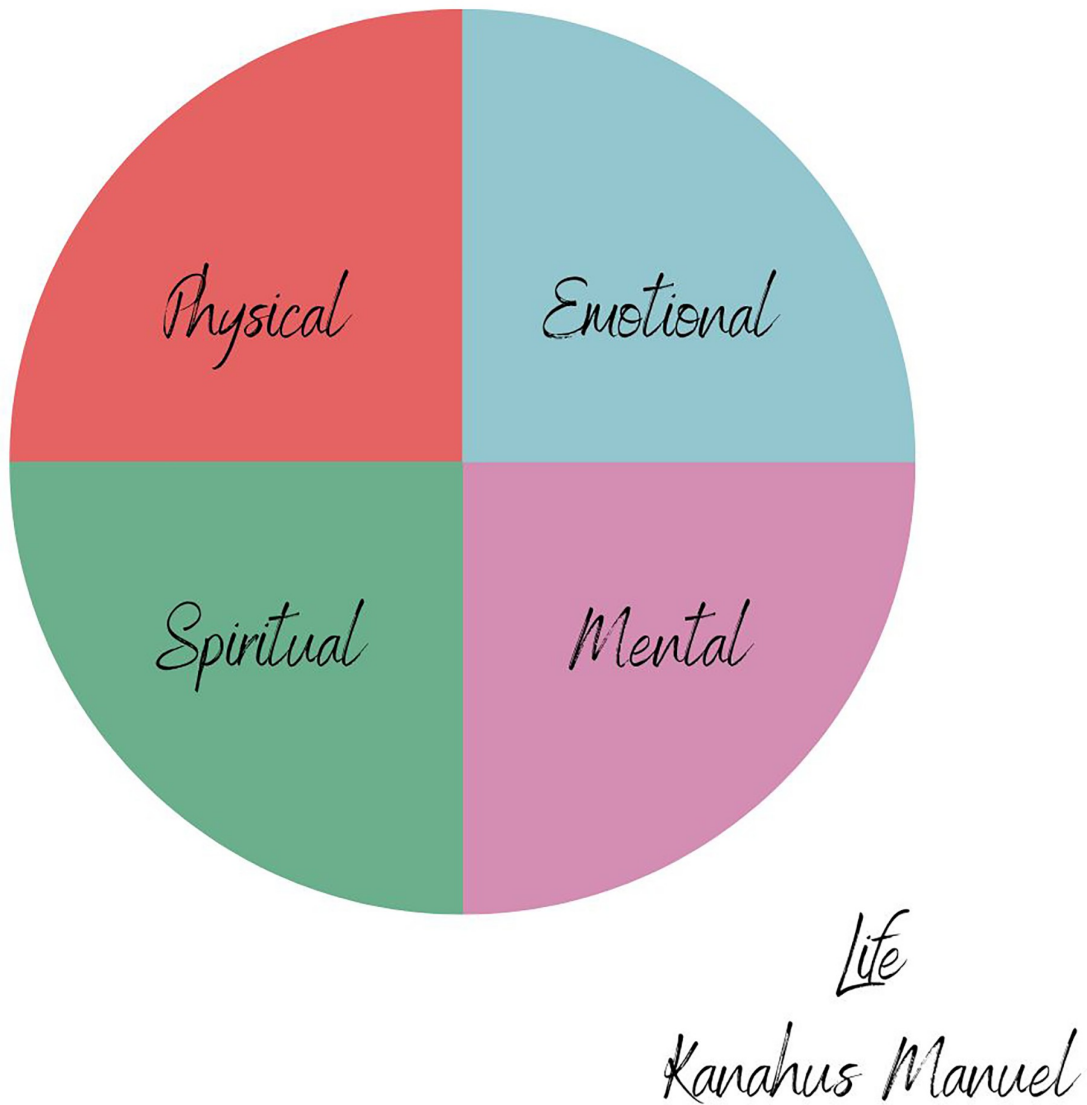
Life by Kanahus Manuel, adapted from the 2021 People’s Health Hearing.

This is closely linked to tunnel vision for health itself, which has tricked us into believing that our health is disconnected from each other and the earth. The definition of health has been disrupted by the powerful forces of colonialism and capitalism, which manifest as an individualistic conceptualisation of health—merely a state of being that is absent from disease determined by individual behaviours and/or biological flaws. Imagine the possibilities created by a health community who challenges this distorted model of health. At the People’s Health Hearing in 2021, Kanahus Manuel conceptualised the various aspects of a healthy life, including physical, emotional, mental and spiritual health [See Fig 2], whilst Paccha Turner Chuji described her Kichwa community’s cosmovision of health based on balanced harmony, respect and equilibrium with Mother Nature [49].

Broadening our understanding of health to one that is collective, political, and ecological is an essential component to tackling the health and climate crises [50]. Advertisements for profitable inhalers to protect individuals from air pollution are common in public places. Instead, calls to join movements for clean air policies which protect everyone’s health, not just privileged individuals, should be the norm. We can only tackle all elements of sustainability when we reclaim sustainability as a social justice issue, instead of the narrow idea of emission reduction. As Fig 1 [2] shows, sustainability has been simplified to carbon emissions, whilst ignoring the role of water, poverty, education, inequality, overconsumption, health, affordable goods and services in living in a sustainable world. We need a health community that acknowledges that it is impossible to be healthy while our neighbour suffers from fuel poverty, when our friends’ homes burn in heat waves, when our farmers’ land is grabbed by monopolies and their families driven to hunger, or when animals die in polluted rivers.

## Economic paradigms for people and planet

Freeing ourselves from tunnel vision enables visualising an expansive and exciting way forward. Not only can an expansive view of health help to spurr climate action, it challenges us to rethink the design of our entire economy. It is the duty of the health community to be a counterforce to the current economic paradigms that make us sick. Health and wellbeing, a basic human right, requires abolishing our current growth-obsessed society that remains in the hands of corporate power. Alternative models that emphasise abundance of wellbeing, such as degrowth [51], doughnut economics [52] and ecosocialism [53] are potential tools to building a better world.

Transforming the economy will require not only a radical change in how resources are governed and distributed, but an overhaul of the fundamental values which underpin the capitalist worldview. For example, as highlighted by the People’s Health Movement Ecosystems and Health Working Group, Luis Maldonado Ruiz outlines how the Indigenous worldview of Sumak Kawsay or Buen Vivir differs from capitalism in fundamental values such as centring collective property, designing institutions around social reciprocity and redistribution, viewing humans as part of nature, equitable access to resources and collective wellbeing [47]. Equitable resource redistribution will require the dismantling of the scarcity mindset, created and maintained by colonial capitalism to justify the artificial scarcity it creates and maintained by carceral systems, such as borders, designed to ‘exclude’ some from access to resources [51]. To build the world we know is possible, we must challenge a deep fundamental belief which has been instilled in so many of us. We must believe that there is enough for everyone to be well.

**Fig 3:**
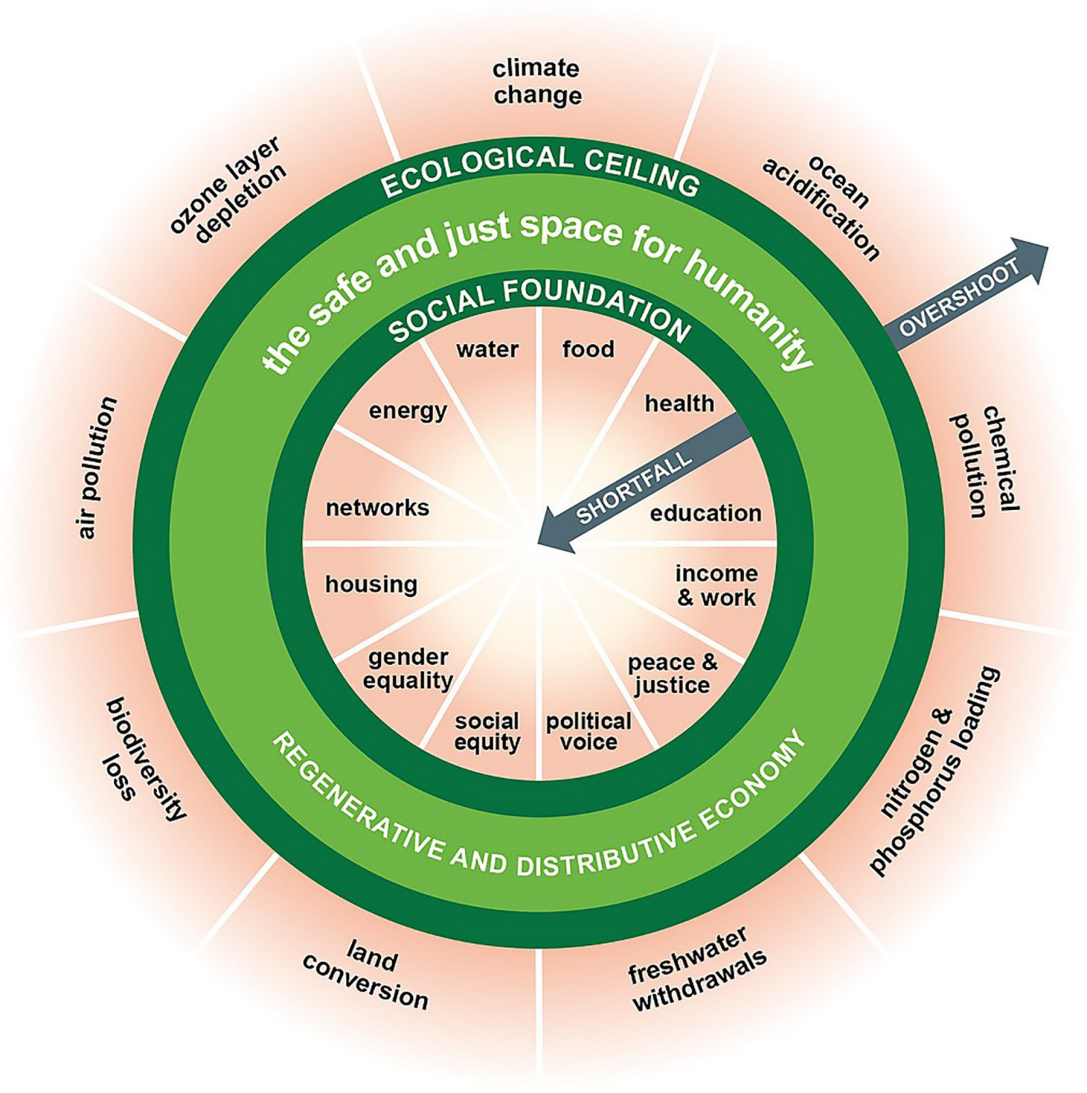
Doughnut economics: A framework for how the economy could develop within a safe environmental space and just social space [52].

Doughnut economics proposes designing societies to achieve a social foundation for human wellbeing (based on the Sustainable Development Goals) without exceeding the ecological ceiling based on planetary boundaries [See Fig 3] [54]. Doughnut economics challenges a fundamental belief that capitalism has instilled in our societies—that there are not enough resources to go around. Instead, it recognises that ecological crises and unnecessary scarcity are created by an economy designed for ‘growth’ and wealth accumulation. Doughnut economics advocates for an economy to be redistributive and regenerative by design so that the essentials of a decent life for all are met, and in doing so, could equitably distribute the social determinants of health.

Ecosocialism is an approach which focuses on class struggle, power, and ownership. Marxist political economy analysis emphasises how power and unequal resource distribution between economic actors shapes the economy and drives exploitation of people and the planet [54]. Importantly, ecosocialism sees ecological harmony as an essential precondition to liberation rather than viewing the environment as simply a source of economic value. Ecosocialist proposals include democratic economic planning; worked owned production; reorienting production to social goods instead of uncontrolled consumption and planned obsolescence; reducing working hours; increasing wages; and implementing wealth redistribution [55]. Challenging power and ownership in the economy to create worker-led, cooperative models of production could have significant health benefits, in particular with regards to economic inequality and production for social and environmental good over profit.

A degrowth approach advocates a managed transition to an economy which maintains an ecological ceiling while prioritising health and social wellbeing. Many of the industries supported in the name of ‘economic growth’, such as the fossil fuel industry, the military and fast fashion, are based on social and environmental violence and need to be dismantled. However, rejecting GDP does not mean rejecting abundance. The goal of degrowth is to move away from an economy designed solely for material output and wealth accumulation. Degrowth advocates for an economy which does not demand endless resource use, but instead distributes resources equitably, and is designed for an abundance of public wellbeing, not private wealth [51]. As Jason Hickel states, “degrowth calls for abundance in order to render growth unnecessary. Degrowth, at its core, is a demand for radical abundance” [56]. Proposals to achieve this include public ownership and dismantling or transformation of harmful industries; wealth and corporate taxation; the implementation of a social foundation including universal public services; a universal basic income; large scale investment in a green transition; and a climate job guarantee [57].

Importantly, for any new economic paradigm to meet the joint goals of health and climate justice, it must implement reparations–this is explicitly part of degrowth. Reparations requires ending economic, social, and political dominance of the global North and returning wealth and land to the communities whose health has been most harmed by colonial capitalism. Reparations are not just a way to address past harm, but a world making project to build just and ecological futures [58].

Reparations are more essential than ever in the context of a climate crisis which will worsen the injustices created by colonialism, slavery, and capitalism. As Táíwò writes—“Climate change will redistribute social advantages in a way that compounds and locks in the distributional injustices we have inherited from history [59].” Formerly colonised parts of the world are at greater risk not just because of their geographical location, but because of unjust wealth and resource distribution.

Reparative justice can take many forms, including unconditional cash transfers; global climate funding in line with responsibility; tackling tax havens; divestment away from harmful industries towards investment in communities; and grassroots democracy [58]. At the international level, Perry proposes that reparations can be materialised through a Global Climate Stabilisation Fund, and a Resilience financing scheme for loss and damage [60], for example. Simultaneously, Heron highlights the importance of food sovereignty, technology transfer, debt cancellation, land back, the end of occupations and the space to develop freely and independently [61].

There is nothing radical about abolishing a system that was built to extract and destroy. It is simply a basic call to value health by ending ongoing harms at their root, repairing damage through monetary and holistic reparations, and building systems with justice and health as their foundation. Colonial capitalism relies on a white supremacist ideology based on hierarchies of power, devaluation of life, and the separation of humans from nature and each other. New economics must also offer social visions based on collective care and transformative justice. *Reparative justice not only includes the transfer of resources and power*, *but of a complete rejection of the values and philosophies which the current economic order is built on*. By leaning on practices that foster values of caring, compassion, and communal healing, we can build a better world, one steeped in abundance of wellbeing, not profit.

## Breaking free from tunnel vision

An individualist, ‘tweak the system’, approach is failing. Without the restraints of tunnel vision, we can identify impactful, justice-driven strategies for climate change and health. Tunnel vision can neither build sufficient power to overcome big oil, nor tackle the root causes of shared struggles, but a politics of solidarity and mass mobilisation against the elites can.

Working in solidarity with other progressive struggles is the only way to achieve the level of power needed to enact change. Mass social movements centred on community organising and protest have elected new governments in Chile and Colombia with pledges to tackle the oppressive systems driving injustice [62]. National strikes in Ecuador by the Indigenous community led the government to ban all new mining projects near water reserves and ancestral Amazonian lands [63]. Strike participants from the UK National Union of Rail, Maritime and Transport workers have linked the cost-of-living crisis, transport, corruption, and climate in a successful communications campaign to garner public support on the strikes [64]. These are examples of actions that have moved beyond their current field of vision, connecting their issue to several others, and mobilising collaboratively.

Leaning on abolitionist discourse provides important understandings that allow us to expand our critical consciousness and reclaim our stolen imagination. First, we can learn lessons about getting to the root of the problem: abolition is about abolishing the conditions under which prisons became solutions to problems. Applying this to climate change and health, it means addressing structural factors such as colonialism, militarism, and capitalism. Second, abolition is both the dismantling of harmful current structures, while also constructing communal social institutions that prevent harm and galvanise collective decision-making. As Ruth Wilson Gilmore states, “Abolition is about presence, not absence. It’s about building life-affirming institutions [65].” Working in partnership with colleagues supporting the abolition of current systems that actively harm individuals and communities, and the creation of alternative systems that centre collective care and well-being, is an exciting opportunity for the climate change and health community [29, 66].

Tunnel vision manifests as the epistemic injustice of whose stories, solutions, and visions are told. Aurora Levins Morales writes about the power of storytelling and listening [67]. Firstly, to understand where we are today outside of our limited individual perspective. Secondly, shared stories generate a vision of the caring, inclusive future we want. It is critical to ask whose stories and solutions are missing from my work. In the health community, the voices of global North ‘experts’ often dominate.

There are several organisations in the UK, where we are based, breaking free from tunnel vision. Transforming ways of working requires deep humility and curiosity, and we invite you to ask what you can learn from others in our journey towards a flourishing and healthy world. Each movement below operates in different spaces with a range of methods. They each place justice at the centre of all efforts, taking a solidarity-driven approach, with accountability lying with the communities most impacted by injustice.

Through review of the current discourse and discussions with colleagues, we present a non-exhaustive list of organisations and movements in the UK climate change and health space that are breaking free from tunnel vision (Table 1). This list contains a description of their work, with key considerations to be used by members of the health community when participating in climate change and health work. Some of the groups listed, such as the People’s Health Movement, Post-Extractive Futures and the People’s Health Hearing are international—building strategy across shared struggles, exchanging learning across contexts and facilitating solidarity-based organising between the global North and the global South. Current structures are not providing a platform or sufficient resourcing for the efforts made by some of these groups that are critical in moving towards an expansive climate and health justice. We honour them and call upon the health community to know their names, learn about their way of working, and support their work. We encourage readers to explore their own local contexts, as well as how their context may connect through exploitation, solidarity, or history to struggles in other geographies.

**Table 1:** Organisations and movements breaking free from tunnel vision within the climate change and/or health space (UK and international).

Name of organisation or movement	Description of work	Key considerations for the health community
The Centric Lab	A research lab building fair systems for data that communicate environmental and health justice. They use neuroscience, ecological research, social justice principles and geospatial data, to understand how the places we live impact our health. They recognise that justice comes from a strong local voice. All communities should have strong representation and influence in local government.	How can we use health justice to approach research, frame the science, the type of work that is chosen, and how the work is done? We must acknowledge that knowledge of Indigenous Peoples is essential to move towards good health of people and the planet. We must recognise that science and scientists are to serve people and communities.
Civic Square	A group creating spaces for communities to imagine and build alternative economic, social, and ecological paradigms. They are a public square, collectively owned and built 21st Century civic and social infrastructure, providing a place for communities to gather and connect. They also provide a neighbourhood economics lab, focused on exploring, experimenting, and testing and building resilient, regenerative neighbourhoods. It is rooted locally and connected globally through partners and projects. They encourage a creative and participatory ecosystem.	How can we create safe, creative, and exciting physical spaces for communities to explore acting for large scale system change, whilst also dreaming and imagining alternative realities?
Climate Reparations UK	A collective of social and climate justice groups demanding reparative justice in many forms: the UK and polluting industries paying for their global climate harms and giving decision making power to frontline communities experiencing health injustice.	How can we work collectively with other social justice and health groups to push for bold action on climate reparations? How can the health community mobilise to implement climate reparations urgently needed for the health harms spread unfairly across the world?
Cradle Community	A collective of organisers committed to radical education and building understanding of prison abolition and transformative justice. They connected climate action and abolition in their latest book.	How can we draw connections across social justice movements with a shared ethic of health justice?
Decolonising Economics	Strategists aiming to move power and resources to those who are building a just transition through deepening the analysis of colonial economic and financial systems. They work alongside partners and allies to facilitate a solidarity economy and invest in the leadership of marginalised communities of colour who are organising to towards community ownership of assets, as a strategy for reparations that centres collective healing.	What is our role in building a solidarity economy rooted in racial justice principles? How can we divest from whiteness and create the infrastructure that organises those with wealth to redistribute towards marginalised communities who are investing in the solidarity economy.
Doctors for Extinction Rebellion	A grassroots group pushing the boundaries of what it means to protect the health of patients, facing arrest outside the UK Treasury and JP Morgan offices to protest fossil fuel financing.	How can we use mass civil disobedience to fight for health and climate? How can we support our colleagues to take direct action and protest safely? How can we highlight the role of the state and police in protecting fossil fuel interests?
Envisioning Environmental Equity Collaborative	A joint effort of organisations in Brazil, the Philippines, the UK, and Uganda that takes anti-oppression as the starting point for solutions for environmental and health equity, placing most affected people and areas at the heart of the narrative. They use film, comics, podcasts, webinars, and more to explore the nexus of climate change, racism, and health.	How can we partner with most affected people and areas, centring their solutions, making space and platforming their visions for climate and health equity? How do we ensure that all solutions, research, activism, and education are anti-racist?
Forensic Architecture	A research group that works in partnership with institutions across civil society, from grassroots activists to legal teams, to international NGOs and media organisations to undertake research on behalf of communities affected by various, interconnected injustices. Findings from their investigations have been presented in national and international courtrooms, parliamentary inquiries, and exhibitions at some of the world’s leading cultural institutions and in international media, as well as in citizen’s tribunals and community assemblies.	How can research and advocacy be conducted in partnership with a wide range of actors? We must connect various injustices such as conflict, police brutality, border regimes and environmental violence, as having one common root cause—colonial capitalism. How can climate and health research be truly multidisciplinary, disrupt power structures and be utilised and exhibited in local, national, and international arenas to demand change?
Healing Justice London	A collective building community-led health & healing. Reimagining health systems—to emerge from a period of discovery, imagination, and collective growth together with ideas for a just and equitable system of community-centred health. They use intersectional and multi-layered and systems approach to disarm the cycles of harm, ill-health, and chronic unsustainability that oppression reproduces in communities and social justice movements.	How can we empower communities that are marginalised to lead, shape, and determine health and well-being? How can we help to build radical alternative public health approaches to climate and health?
Health for a Green New Deal	A campaign by Medact mobilising health workers working towards a transformative Green New Deal in the UK. The growing movement for a Global Green New Deal represents an intersectional and whole-systems-oriented approach to climate action that puts addressing inequity at its core; recognising that climate, health, social and economic justice can only be achieved together. Public engagement on climate, health and social justice issues take place across the country via ’Climate Clinics’, where health workers communicate aspects of the Green New Deal to the public in a consultation format.	How can the health community build power to overcome corporate interests through strategic targeting of national policy, and contribute to political mobilisation at the national level, which is part of a wider international movement?
Land Body Ecologies	A global interdisciplinary network exploring the deep interconnections of mental and ecosystem health. Their research is rooted within communities at the forefront of today’s climate, ecosystem, and land rights issues. They work in collaboration to seek to understand the traumas endured when the land suffers.	How can we engage with creative partners to help communicate expansive climate and health solutions? How do we communicate the importance of our body and mind’s connection with the land when advocating for change?
Land In Our Names	A grassroots Black-led collective that mobilises people to support the redistribution of land to communities of Black people and people of colour in Britain, disrupting the colonial legacies of land ownership, and using land as a site of healing and repair for the various traumas carried by minoritised people.	How can we use our voice, skills, and capacity to facilitate returning wealth and land to the communities whose health has been most harmed by colonial capitalism?
People’s Health Hearing	The People’s Health Hearing is a co-created deep listening and testimony forum for frontline communities facing the violence of extractive industries. The forum highlights the colonial nature of industries such as fossil fuels and mining and builds solidarity across movements in the global South and between movements in the global South and the global North.	How can we centre the voices of those most impacted by decades of extractive industry violence? How can we highlight the root causes of the climate crisis in colonial resource capture? How can we demand reparations for the harm done to communities on the frontlines of extractivism? How can we build meaningful solidarity across our movements?
People’s Health Movement	The People’s Health Movement is a global collective of health workers, activists, academics and communities organising for health justice, as set out by the principles of the People’s Charter for Health. Their ecosystems and health global circle are challenging all extractive industries that harm health, and building collaborations between global North and South resistors of extractivism.	How do we challenge the capitalist model of ’development’ which relies on extractivism? How do we build visions of progress which see ecosystems not as sources of economic value but as the foundations of health? How do we challenge the idea of ’health’ to go beyond health systems, but the right to health in its entirely?
Post-Extractive Futures	Post-Extractive Futures is a space for mutual learning and solidarity-weaving amongst activists dreaming, enacting, and mobilising toward postcapitalist, ecological, feminist, afro-diasporic, intergenerational, indigenous and decolonial futures. The goal of this space is to share experiences, lessons learned, cautions, recommendations, provocations, and inspirations.	How can we draw on ideas of climate justice, just transitions, Green New Deals, Eco-Social Pacts, abolition, and degrowth, to imagine what world to build? How can we build solidarity across borders?
Stop Cambo	A campaign that undertakes mass mobilisation, direct confrontation of politicians, and targeting oil companies. It is successfully delaying and challenging the expansion of oil fields in the UK.	How can we strategically target harmful industries? How can we use media and communications to debunk fossil fuel propaganda? How can we mobilise the public against fossil fuels?
